# Randomized, crossover clinical efficacy trial in humans and mice on tear secretion promotion and lacrimal gland protection by molecular hydrogen

**DOI:** 10.1038/s41598-021-85895-y

**Published:** 2021-03-19

**Authors:** Miyuki Kubota, Motoko Kawashima, Sachiko Inoue, Toshihiro Imada, Shigeru Nakamura, Shunsuke Kubota, Mitsuhiro Watanabe, Ryo Takemura, Kazuo Tsubota

**Affiliations:** 1 Department of Ophthalmology, Keio University School of Medicine, 35 Shinanomachi, Shinjukuku, Tokyo, 160-8582 Japan; 2 Department of Ophthalmology, Shonan Keiiku Hospital, Kanagawa, Japan; 3 Graduate School of Media and Governance, Keio University, Kanagawa, Japan; 4 Hanegino Mori Eye Clinic, Tokyo, Japan; 5 Clinical and Translational Research Center, Keio University Hospital, Tokyo, Japan; 6 Tsubota Laboratory, Inc., Tokyo, Japan

**Keywords:** Molecular biology, Medical research

## Abstract

The incidence of dry eye disease is increasing worldwide because of the aging population and increasing use of information technology. Dry eye disease manifests as tear-layer instability and inflammation caused by osmotic hypersensitization in tear fluids; however, to our knowledge, no agent that treats both pathologies simultaneously is available. Molecular hydrogen (H_2_) is known to be effective against various diseases; therefore, we aimed to elucidate the effects of H_2_ on tear dynamics and the treatment of dry eye disease. We revealed that administering a persistent H_2_-generating supplement increased the human exhaled H_2_ concentration (*p* < 0.01) and improved tear stability (*p* < 0.01) and dry eye symptoms (*p* < 0.05) significantly. Furthermore, H_2_ significantly increased tear secretion in healthy mice (*p* < 0.05) and significantly suppressed tear reduction in a murine dry eye model (*p* = 0.007). H_2_ significantly and safely improved tear stability and dry eye symptoms in a small exploratory group of 10 human subjects, a subset of whom reported dry eye symptoms prior to treatment. Furthermore, it increased tear secretion rapidly in normal mice. Therefore, H_2_ may be a safe and effective new treatment for dry eye disease and thus larger trials are warranted.

## Introduction

Dry eye disease is a multifactorial disorder of the ocular surface characterized by a loss in the homeostasis of the tear film and accompanied by ocular symptoms, in which tear film instability and hyperosmolarity, ocular surface inflammation and damage, and neurosensory abnormalities have etiological roles^1^. Currently, dry eye disease affects approximately 5–50% of the world’s population^1^. Dry eye disease affects working performance as well; in a prior study, approximately 65% of office workers were diagnosed either with dry eye or probable dry eye disease^2^. Further, a 4.8% labor productivity loss due to dry eye disease has been recorded, approximately the same as caused by migraine^2^. This condition has also affected both happiness and sleep quality^3,4^.

The number of patients with dry eye disease is expected to rapidly increase in the future because of the growing size of the older adult population and changes in lifestyle influenced by the widespread use of information technology apparatus^5^. The reported cause of dry eye disease is a decrease in the amount of tear production due to aging, wearing contact lenses, and metabolic syndromes such as dyslipidemia, hypertension, or hyperglycemia with abdominal obesity^6^. The two major contributors to dry eye disease are aqueous deficiency and evaporative dry eye disease^1^. The main treatment for dry eye disease in Japan is supplemental teardrops for the stabilization of the tear layer. However, cyclosporine eye drops, which are immunosuppressive agents, are reportedly effective according to a United States study^7^. Given the aforementioned imminent projected increase in the number of patients with dry eye disease, a fundamental treatment method that can address both major pathologies is urgently needed.

Molecular hydrogen (H_2_) has long been used to prevent decompression sickness^8^, since it is unlikely to form bubbles owing to its tissue solubility, diffusivity, and reactivity properties within the body^9^, and H_2_ is highly safe^10^. Since the molecular weight of H_2_ is minimal, it rapidly diffuses to the site of oxidative stress and has antioxidant and anti-apoptotic properties^11^. H_2_ selectively reduces cytotoxic reactive oxygen species (ROS), such as hydroxyl radicals, and preserves the ROS needed by living organisms^11^.

The efficacy of H_2_ has been reported in inflammatory models, including hepatitis^12^ and pancreatitis^13^; in ischemia–reperfusion injury models, including those for the nerves^14^, liver^15^, kidney^16^, myocardia^17^, and small intestine^18^; and in an alkali-burn model to investigate the suppression of corneal angiogenesis^19^. In clinical studies, the efficacy of H_2_ for alleviating oxidative stress, suppressing inflammation, improving metabolism, and treating diabetes^20^, hyperlipidemia^21^, mitochondrial myositis^22^, and rheumatoid arthritis^23^ has been suggested^24^.

Recently, it has been demonstrated that milk consumption yields H_2_ in the breath^25^. Furthermore, our group previously reported on milk-producing capability of H_2_, which is created by adding galacto-oligosaccharide, maltitol, and glucomannan (the active ingredients for intestinal H_2_ production^26^) to a solution comprising cow milk (50%) and skim milk (50%)^27^. This milk induced the intestinal microbiota to produce H_2_, preventing the decrease in tear stability that occurs when adult human participants use visual display terminals daily^27^. However, there are no reports on the direct action of H_2_ on the endocrine glands. The purpose of this study was to elucidate the effects of H_2_, a safe and inexpensive therapy, on the external secretory function of the lacrimal glands and to investigate whether H_2_ protects the eye from tear loss in a severe dry eye model.

## Results

### Clinical study

#### Participant characteristics

Table 1 shows the characteristics of the participants in the clinical study. The mean age of the participants was 35.3 ± 4.16 years (age range 29–41 years).

**Table 1:** Characteristics of the participants in the clinical study.

Sex	Age (years)	Smoking	Contact lens
Female	36	No	No
	32	No	No
	41	No	No
	37	No	No
Male	29	Yes	No
	29	Yes	Yes
	35	Yes	No
	38	Yes	No
	36	No	No
	40	No	Yes
Total	10	4	2
Mean ± SD	35.3 ± 4.16		

#### Exhaled H_2_ concentration

**Figure 1:**
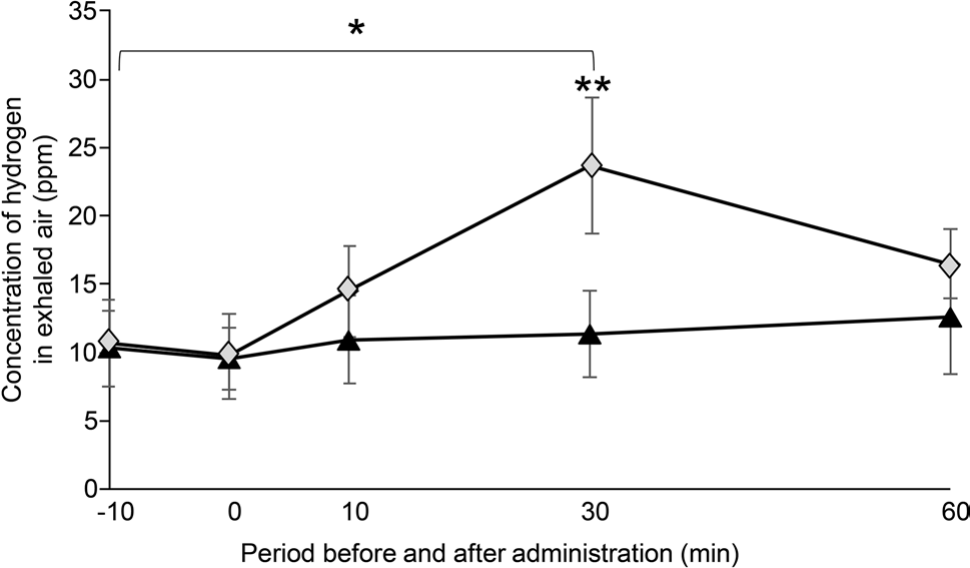
Changes in the molecular hydrogen (H_2_) concentration in exhaled air. All results are expressed as the mean ± standard error. Significant differences from the control group are indicated by *p < 0.05 or **p < 0.01*.* Black triangle: control, grey diamond: SUPER H2 (H_2_).

The exhaled H_2_ concentration was significantly higher at 30 min after SUPER H2 administration than it was at 30 min after mineral water administration (*p* = 0.006); moreover, it was significantly higher at 30 min after SUPER H2 administration than it was at the pre-SUPER H2 administration time points (*p* = 0.013) (Fig. 1). The changes in the exhaled H_2_ concentration for each of the 10 participants are shown in Supplementary Fig. S1.

#### Effect of SUPER H2 on objective ophthalmological examinations

**Figure 2:**
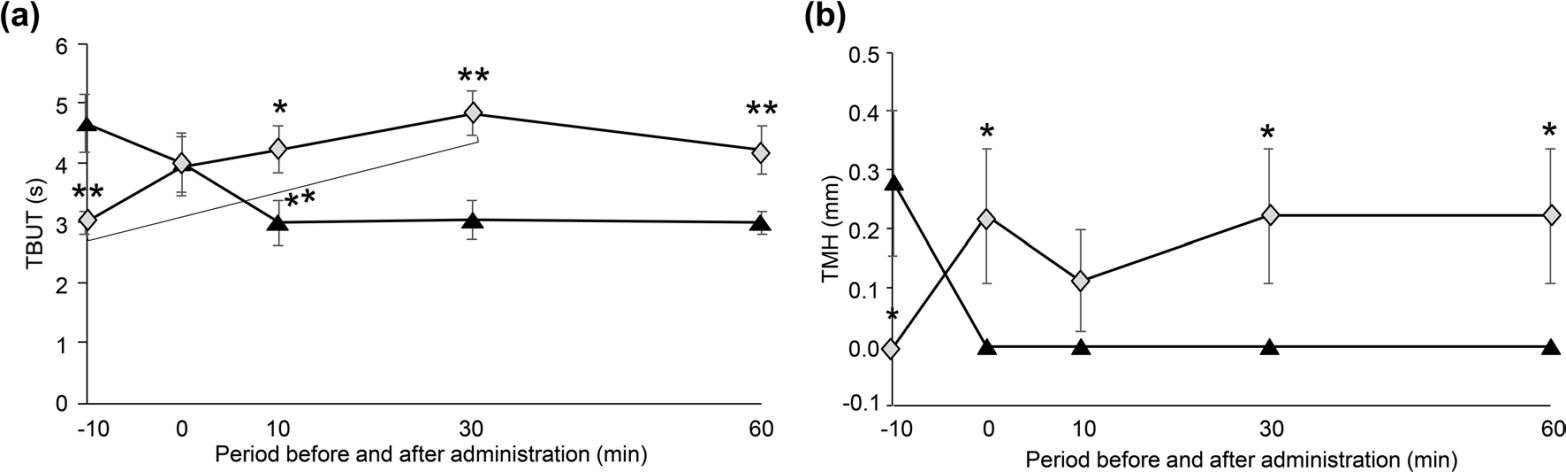
Changes in the tear film breakup time (TBUT) (**a**) and tear meniscus height (TMH) (**b**) over time. All results are expressed as the mean ± standard error. Significant differences from the control group are indicated by *p < 0.05 or **p < 0.01*.* Black triangle: control, grey diamond: SUPER H2 (molecular hydrogen; H_2_).

The tear film breakup time (TBUT) at 30 min was significantly higher after SUPER H2 administration than it was after mineral water administration (*p* = 0.00041); moreover, it was significantly higher 30 min after vs. before SUPER H2 administration (*p* = 0.00000000006) (Fig. 2a). Similar to the TBUT, the tear meniscus height (TMH) was significantly elevated at 30 and 60 min after administration in the SUPER H2 group compared to that in the control group (both *p* = 0.042) (Fig. 2b).

#### Assessment of subjective ocular symptoms

**Figure 3:**
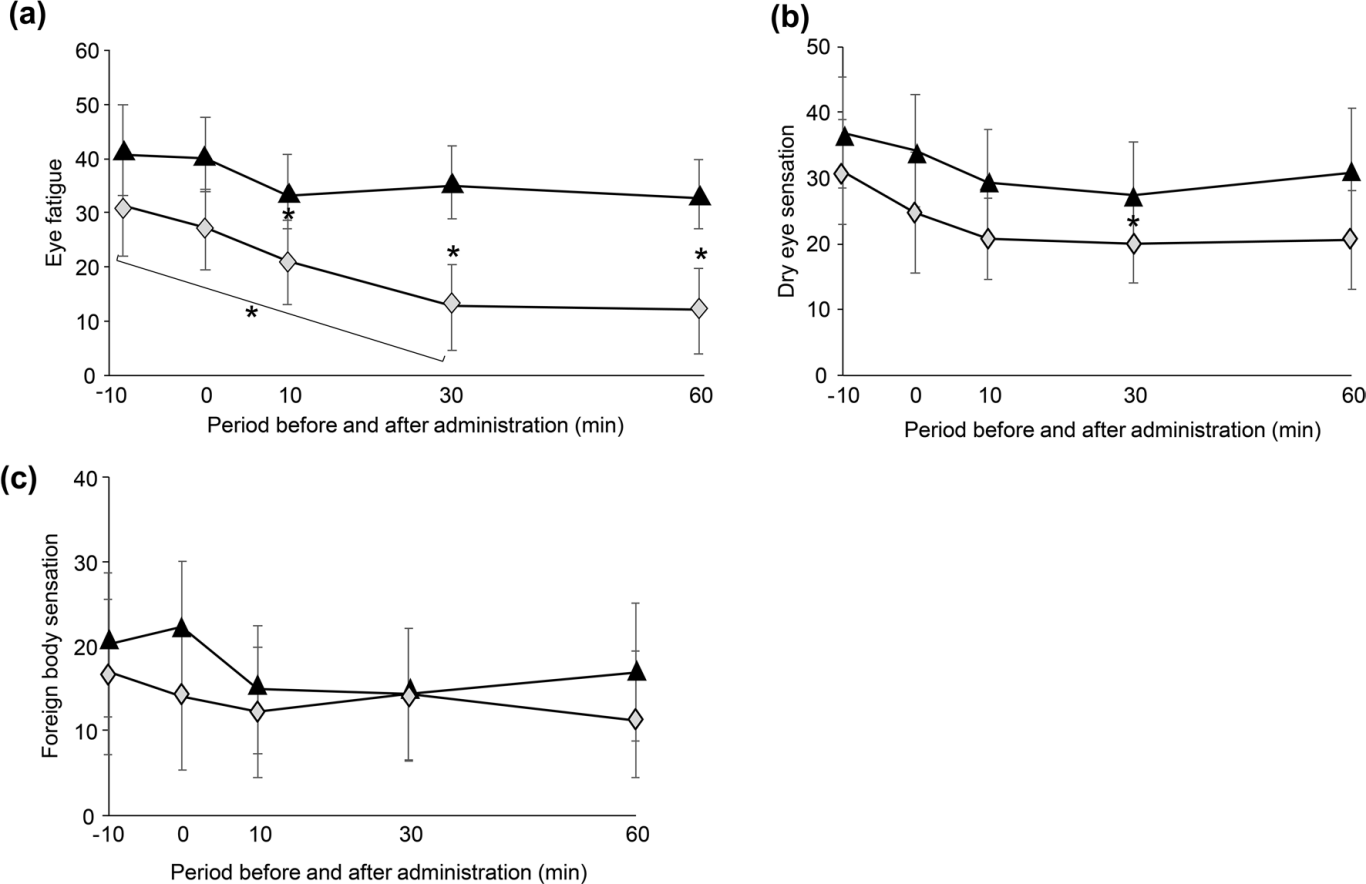
Changes in the subjective ocular symptoms of eye fatigue (**a**), dry eye sensation (**b**), and foreign body sensation (**c**) over time. All results are expressed as the mean ± standard error. Significant differences from the control group are indicated by *p < 0.05*.* Black triangle: control, grey diamond: SUPER H2 (molecular hydrogen; H_2_).

Among the subjective symptoms, ocular fatigue was significantly lower 30 min after SUPER H2 administration than it was 30 min after water administration (*p* = 0.014); moreover, it was significantly lower 30 min after SUPER H2 administration than it was at the pre-SUPER H2 administration time points (*p* = 0.013) (Fig. 3a).

Dry eye sensation was significantly lower at 30 min after SUPER H2 administration than it was at 30 min after water administration (*p* = 0.049) (Fig. 3b). There was no significant difference between the SUPER H2 and control groups regarding foreign body sensation (Fig. 3c).

#### Side effects

No side effects from the ingestion of the 10 SUPER H2 capsules were observed or reported by the participants.

### Animal study

#### Effect of SUPER H2 on tear secretion capacity in non-stressed mice

**Figure 4:**
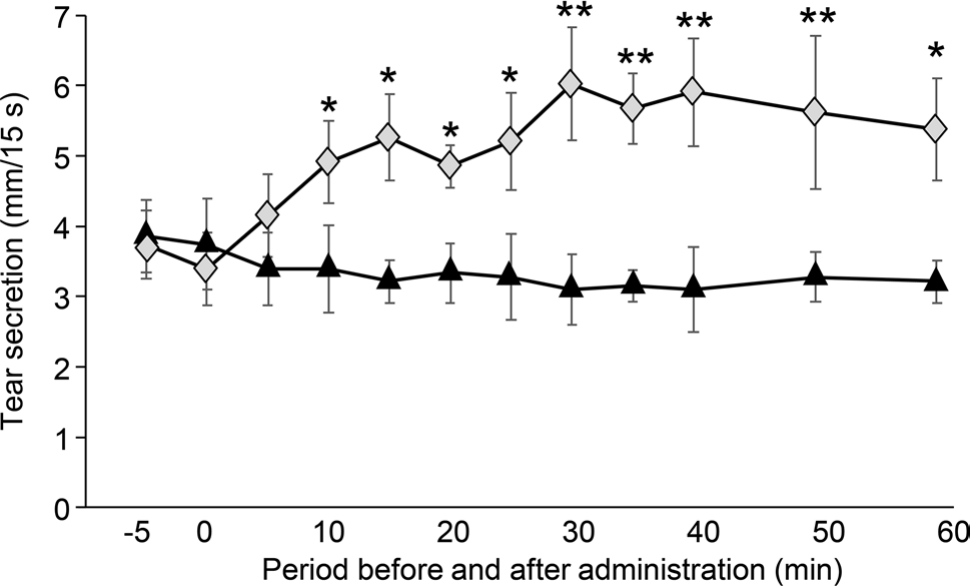
Effect of molecular hydrogen (H_2_) on the tear secretion in non-stressed mice. All results are expressed as the mean ± standard deviation (n = 5). Significant differences from the vehicle group are indicated by *p < 0.05 or **p < 0.01*.* Black triangle: vehicle, grey diamond: SUPER H2 (H_2_).

SUPER H2 was administered to the mice; the tear secretion at 10 min after administration was significantly greater in the SUPER H2 group than it was in the control group (Fig. 4). The significant differences maximum duration was 60 min (*p* = 0.039, 0.029, 0.013, 0.011, 0.003, 0.003, 0.001, 0.003, and 0.012 for 10, 15, 20, 25, 30, 35, 40, 50, and 60 min, respectively).

#### Evaluation of SUPER H2 in a murine stress-induced dry eye model

**Figure 5:**
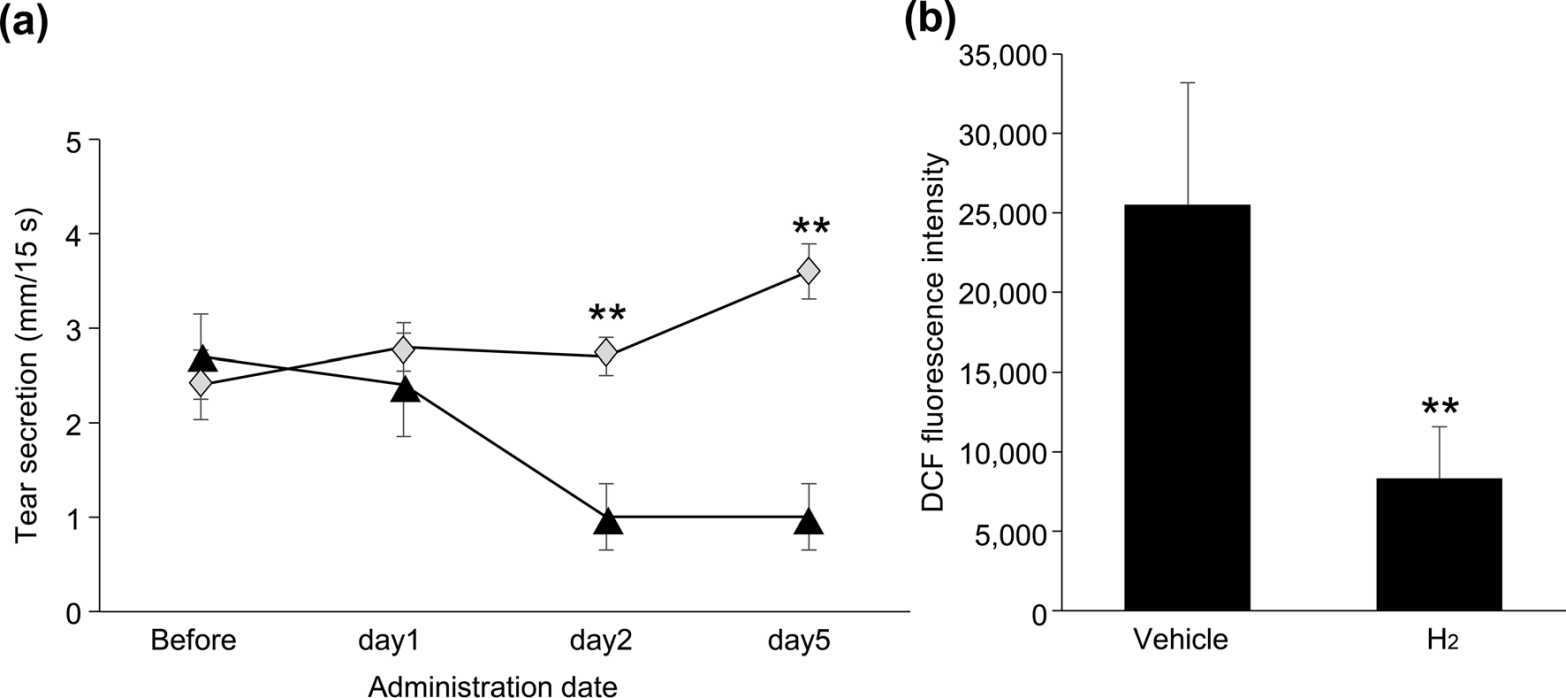
Evaluations of the stress-induced dry eye murine model. (**a**) Measurement of tear secretion using the average value for the left eyes in the stress-induced dry eye model. (**b**) Quantitative measurement of the reactive oxygen species in the lacrimal gland in the stress-induced dry eye model. All results are expressed as the mean ± standard deviation (n = 5). Significant differences from the vehicle group are indicated by **p < 0.01*.* *DCF* dichlorofluorescein. Black triangle: vehicle, grey diamond: SUPER H2 (molecular hydrogen; H_2_).

H_2_ supplementation significantly suppressed the reduction in tear secretion that was observed for the control group in the mouse stress-induced dry eye model on day 2 and day 5 (both *p* = 0.007) (Fig. 5a). There was no significant difference in the amount of food eaten or in the body weight in each group (Supplementary Table S1).

#### Determination of ROS in lacrimal gland tissue after stress induction

The SUPER H2 group showed a significant reduction in ROS fluorescence intensity in the lacrimal gland tissue compared to that in the control group (*p* = 0.00016) (Fig. 5b).

## Discussion

Our study found that administering a safe and inexpensive H_2_ supplement affected the external secretory function of the lacrimal glands and protected the eye against tear loss in the severe dry eye model. Tear fluids are composed of an oil layer, a liquid phase, and a mucin layer and have important functions such as preventing drying of the eyes, preventing infection via a cleaning action, correctly refracting light by smoothing the eye surface, and supplying oxygen and nutrients. Eye blinking assists with tear secretion since it maintains a tear coating of a certain thickness on the eye surface. Therefore, reduction in tear secretion causes vision loss and symptoms such as pain and itching. This can also lead to an infection of the ocular surface.

Dry eye disease greatly affects the quality of life, and is common in office workers, especially visual display terminal workers; thus, active management of this medical condition is urgent. Although it has been shown that oxidative stress is deeply involved in both aging and its pathogenesis of many age-related diseases^28^, dry eye disease has also been reported to be deeply involved in the pathology of oxidative stress^29,30^.

The results of this study suggest that H_2_ is effective as a treatment for aqueous deficiency and evaporative dry eye disease, which are the two major conditions in dry eye disease. Approximately 300–1000 mL of H_2_ is produced in the body, every day by intestinal bacteria, and it is a familiar and safe substance that is also discharged during breathing. We confirmed that a single dose of SUPER H2 increases the concentration of H_2_ in the breath in humans. Not only is H_2_ able to selectively reduce the highly cytotoxic ROS^11^, but it is also indirectly considered an antioxidant owing to its scavenging properties; moreover, it is known to exhibit anti-stress properties, and is reported to result in various pathological improvements due to its anti-apoptotic effects^24^.

The increase in the intracellular signaling substances Ca^2+^ and cyclic adenosine monophosphate (cAMP) in exocrine gland cells is closely related to the secretion of digestive enzymes^31^. The mechanism underlying Ca^2+^ mobilization is the binding of the neurotransmitter to receptors. The resulting inositol triphosphate acts on the receptors on the vesicle membrane and promotes Ca^2+^ release from the vesicles^32–34^. In lacrimal cells, acetylcholine is secreted upon physiological stimulation, and Ca^2+^ ions act as a second messenger. In lacrimal gland cells, there are three types of ion channels: (1) K^+^ channels, (2) Cl^−^ channels, and (3) non-selective cation channels^35^. All of these are activated by Ca^2+^, with decreasing sensitivity in the aforementioned order^35^. The inflow of Cl^-^ caused by the opening of the Cl^−^ channel is accompanied by Na^+^ and physiologically observed as the secretion of water. Thus, Ca^2+^ plays a major role in the secretion of water in the lacrimal glands^35–37^. However, recently, H_2_ has been reported to inhibit inflammatory transcription factors, such as a nuclear factor of activated T cells and cAMP response element-binding protein, by suppressing Ca^2+^ inflow into the cells^38^. As such, H_2_ inhibits the expression of various genes by activating downstream transcription factors^38^. Since this phenomenon of suppressing Ca^2+^ is contrary to the mechanism that increases tear secretion in the acinar cells of the lacrimal gland, further investigation of the dynamics of intercellular Ca ion concentration [Ca^2+^]i^39^ by hydrogen is needed.

In this study, H_2_ suppressed tear reduction and ROS in the lacrimal glands in a mouse stress-induced dry eye model on day 5. It is important to note that most conditions of dry eye in human beings are caused by chronic diseases and cannot be compared with an acute murine severe dry eye model. However, tear secretion was significantly greater at 10 min after SUPER H2 administration than it was at 10 min after vehicle administration in normal non-stressed mice. This effect of increasing tears in normal mice may indicate effectiveness for dry eye resulting from a chronic disease.

The promotional effect of H_2_ on tear secretion that occurred a short time after administration is a phenomenon that cannot be explained because of changes in gene expression and signal induction, or by the scavenging of H_2_ by intestinal microbiota, as previously reported^12,40^. The reason for the enhanced tear secretion may be due to another mechanism that does not involve oxidative stress. The lacrimal gland is the representative external secretory gland; tear secretion is normally enhanced by the endocrine function of the acinar cells. The mechanisms underlying the augmented tear secretion that we observed following H_2_ administration should be investigated in the future.

Our study has several limitations. The small sample size was both due to its exploratory nature and the difficulty in enrolling a large number of participants. Other possible substantial effects could not be detected because of the small sample size. A similar study with more participants should be performed to confirm and expand upon our findings. After 30 min of SUPER H2 administration, the hydrogen concentration in the exhaled air began to decline; thus, longer observation periods are required. A single dose of SUPER H2 was administered in this study, but it is necessary to observe the H_2_ concentration in exhaled air and the dry eye symptoms of multiple administrations of SUPER H2.

In conclusion, our small exploratory study revealed that H_2_ significantly and safely improved tear stability and dry eye symptoms in 10 adult human subjects, and improved tear secretion from the lacrimal glands in a severe dry eye model. The two major pathologies of dry eye disease are aqueous deficiency and evaporative dry eye disease. Thus far, only therapeutic agents that exert effects on one of these two pathologies have been developed and used. To our knowledge, the present study is the first to show that H_2_ is likely to affect both pathologies simultaneously. Since H_2_ is safe and inexpensive, its clinical application for dry eye symptoms should be further examined in large-scale investigations.

## Methods

### Clinical study

#### Ethics

This study was performed in compliance with the guidelines of the Declaration of Helsinki (amended in 2008). The study protocol was approved by the Ethical Review Board of Haneginomori Eye Clinic (approval no.: 19004). The trial (UMIN000037169) was registered in the University Hospital Medical Information Network in Japan on June 25, 2019.

All participants received a full explanation of the study protocol, and they provided written informed consent before enrollment. To ensure privacy, all records were identified using an anonymized participant identification number. All participants were permitted to withdraw from the study at any time (for any reason) if needed.

#### Study design

This was a prospective, randomized, placebo-controlled, crossover study designed to evaluate SUPER H2-induced changes in the exhaled H_2_ concentration in adult volunteers. The study was performed between June 14, 2019, and August 31, 2019, at the Haneginomori Eye Clinic in Tokyo, Japan, and was registered with the University Hospital Medical Information Network in Japan (UMIN000037169).

#### Participants

The enrolled participants were 10 adult Japanese volunteers, aged 20–50 years.

The following exclusion criteria were set: (1) current or previous severe ocular diseases such as strabismus, cataract, or glaucoma; (2) risk of developing seasonal allergies during the study period; (3) laser-assisted in situ keratomileusis performed within the previous 3 months; (4) allergy to SUPER H2; (5) routine use of multivitamins, mineral supplements, or probiotics, which affect the intestinal environment; (6) current use of medications and/or undergoing chronic medical treatment, including the use of prescription ophthalmic drops; (7) symptoms of constipation; (8) pregnancy; (9) breastfeeding, and (10) underlying diseases during treatment. Some of the human participants may have had mild dry eye because several of the participants reported dry eye symptoms.

#### SUPER H2 interventions

In this two-group crossover study, the active study group was administered a persistent H_2_ generating supplement (SUPER H2; DHC Corp., Tokyo, Japan), whereas the active control group received mineral water (EVIAN; Ito En, Ltd., Tokyo, Japan); SUPER H2 and mineral water were administered to the same participants, on different days, with a crossover period of 1–3 days, in a randomized order. Mineral water (500 mL) was administered either alone or with 10 capsules of SUPER H2; in either case, the water was consumed as quickly as possible (preferably within 5 min).

After SUPER H2 and/or mineral water administration, the exhaled H_2_ concentration was measured over time. In addition, changes in subjective symptoms were determined, and the TBUT and TMH were measured.

To exclude the effects of the participants’ dietary compositions, they were instructed to fast for at least 12 h before SUPER H2 and/or mineral water administration, skipping the evening meal the day before and skipping breakfast on the study date. They were permitted to drink water during the fasting period.

#### *Exhaled H*_*2*_* concentration measurement*

A flute-style exhaled air collection bag (MBA-150; TAIYO Instruments, Inc., Osaka, Japan) was used to collect and store the exhaled air before; immediately upon intake; and 10, 30, and 60 min after ingesting SUPER H2 or mineral water. All samples were analyzed simultaneously for measuring H_2_ concentrations in exhaled air via gas chromatography using the sulfur chemiluminescence detection method (TRIlyser MBA-3000; TAIYO Instruments); 1 mL from the exhaled airbag was injected into the TRIlyser MBA-3000 using a gas-tight syringe.

#### Ophthalmological examinations

An experienced investigator measured the TBUT using a previously reported method^41^. After 2 μL of preservative-free 1% fluorescein dye was placed in each eye, the TBUT was measured three times, and the mean value (combined mean value of both eyes) was obtained. Corneal and conjunctival fluorescein staining scores were graded between 0 (none) to 3 (severe)^41–43^. The ocular examinations were performed before the study and at 4 and 8 weeks after SUPER H2 or mineral water administration. The investigators were not blinded during the study owing to the cross-over nature of the study.

#### Assessment of subjective ocular symptoms

We assessed the following subjective ocular symptoms: eye fatigue, dry eye sensation, and foreign body sensation. The participants were questioned about the symptoms before; immediately upon intake; and 10, 30, and 60 min after administration. The symptoms were graded between 0 and 100 on a visual analog scale.

#### Safety assessments

Participants were instructed to report any adverse events during a medical interview conducted at every visit throughout the study period.

#### Statistical analyses

All results are expressed as the mean ± standard error. For all parameters, paired *t*-tests were used for intragroup comparisons and the *F*-test (followed by Student’s *t*-test when *p* ≥ 0.05 using the *F*-test) was used for intergroup comparisons (H_2_ supplement vs. control). A *p*-value < 0.05 was considered statistically significant. All statistical analyses were performed using PASW Statistics 18 software (SPSS Japan, Inc., Tokyo, Japan).

### Animal study

All experimental procedures were approved by the ethics committee of Keio University and were conducted in accordance with the National Institutes of Health guidelines, Approval No. 19054-(0). Humane animal care conformed to the Association for Research in Vision and Ophthalmology Statement for the Use of Animals in Ophthalmic and Vision Research. The study was carried out in compliance with the ARRIVE guidelines.

Ten female C57BL/6J mice aged 7 weeks were purchased from CLEA Japan, Inc. (Tokyo, Japan). Female mice were arbitrarily chosen to exclude the effects of sex differences and to avoid the risk of mating during the experiment. The mice were housed at a room temperature of 23 ± 2 °C and humidity of 60 ± 10%, with an alternating 12-h light–dark cycle (8 a.m. to 8 p.m.). Food and drinking water were provided ad libitum.

#### Changes in tear after SUPER H2 administration to non-stressed mice

After administering SUPER H2 (57 mg/kg suspended in 5 mL of Milli-Q water) to non-stressed mice, the tear secretion was measured every 5 min for the first 40 min and then every 10 min up to 60 min.

#### Mouse stress-induced dry eye model and SUPER H2 application

We used a mouse stress-induced dry eye model to evaluate the efficacy of H_2_ on dry eye disease and tear secretion. The mouse stress-induced dry eye model was based on that described in a previous report^44^. The mice were physically restrained in a 50 mL plastic conical tube and subjected to a stream of air directed at their heads, at a rate of 0.5–1.0 m/s for 4 h. They were placed individually in cages, with water and food available ad libitum for the remaining time. H_2_ was homogeneously suspended in Milli-Q water (MERCK KGaA, Darmstadt, Germany) by vigorous vortex mixing and orally administered once a day to mice at a dose of 57 mL/kg, prior to stress exposure. Plain Milli-Q water (vehicle) was administered to the control mice.

#### Measurements of aqueous tear secretion

A phenol red thread (ZONE-QUICK; Showa Yakunin Kako Co., Ltd., Tokyo, Japan) was placed on the medial canthus for 15 s; the thread length that was discolored brown due to the penetration of tears was measured with an accuracy of 0.5 mm.

The mice were exposed to a stimulus (stress) between day 1 and day 4. Tear measurements were performed on day 0 (without stress), day 1, day 2 (before exposure to stress), and day 5 (the day after stress exposure ended). Five mice were used for each group (the vehicle and the SUPER H2 groups). The left eyes were used for the analysis.

#### Intracellular ROS assay in lacrimal glands

After 4 days of stress exposure, the mice were euthanized with an overdose of anesthesia on day 5. The lacrimal gland tissue was removed for a 2′,7′-dichlorofluorescein diacetate (2′,7′-DCFH-DA) assay. The homogenized cell suspension was obtained from lacrimal gland tissue that was crushed using a crusher while being ice cooled. ROS were measured using the 2′,7′-DCFH-DA assay. The cells were incubated with 10 μM 2′,7′-DCFH-DA for 60 min at 37 °C and subsequently washed with phosphate-buffered saline. The fluorescence intensity was detected using Synergy 4 with a plate reader (BIOTEK INSTRUMENTS, Inc., Winooski, VT, USA) at an excitation wavelength of 485 nm and an emission wavelength of 528 nm.

#### Statistical analyses

All results are expressed as the mean ± standard deviation. Statistical analyses were performed using the Mann–Whitney U test. All analyses were conducted using SPSS version 25 for Windows (IBM Corp., Armonk, NY, USA). A *p*-value of < 0.05 was considered statistically significant.

## Supplementary Information

Supplementary Information.

## Data Availability

All data generated or analyzed during this study are available from the corresponding author upon reasonable request.
