# Supplementary material for: Randomized, crossover clinical efficacy trial in humans and mice on tear secretion promotion and lacrimal gland protection by molecular hydrogen
Source: Sci Rep. 2021 Mar 19;11:6434. doi: 10.1038/s41598-021-85895-y (PMC7979688; doi:10.1038/s41598-021-85895-y)
Supplement: Supplementary file 1 — Supplementary Information. [file 41598_2021_85895_MOESM1_ESM.docx]

**Randomized, crossover clinical efficacy trial in humans and mice on tear secretion promotion and lacrimal gland protection by molecular hydrogen**

Miyuki Kubota,^1,2,3,†,*^ Motoko Kawashima,^1,†^ Sachiko Inoue,^1,4^ Toshihiro Imada,^1^ Shigeru Nakamura,^1^ Shunsuke Kubota,^1,2,3^ Mitsuhiro Watanabe,^1,3^ Ryo Takemura,^5^ & Kazuo Tsubota,^1,6,*^

^1^ Department of Ophthalmology, Keio University School of Medicine, Tokyo, Japan

^2^ Department of Ophthalmology, Shonan Keiiku Hospital, Kanagawa, Japan

^3^ Graduate School of Media and Governance, Keio University, Kanagawa, Japan

^4^ Hanegino Mori Eye Clinic, Tokyo, Japan

^5^ Clinical and Translational Research Center, Keio University Hospital, Tokyo, Japan

^6^ Tsubota Laboratory, Inc., Tokyo, Japan

^†^Miyuki Kubota and Motoko Kawashima contributed equally to this work.

***Corresponding authors:** Miyuki Kubota MD, PhD and Kazuo Tsubota, MD, PhD,

Department of Ophthalmology, Keio University School of Medicine, 35 Shinanomachi, Shinjukuku, Tokyo 160-8582 Japan. Tel: +81-3-3353-1211. Fax: +81-3-3358-5961.

E-mail addresses: [myu.kubota@keio.jp](mailto:myu.kubota@keio.jp); tsubota@z3.keio.jp

**Supplementary Information**

**Supplementary Table S1.** Body weight and daily food intake of the mice

|  | **Body weight (g)** | | |  |
| --- | --- | --- | --- | --- |
|  | **Day 1** | **Day 4** | **Day 5** | **Daily food intake (g)** |
| **Vehicle** | 16.48 ± 0.85 | 17.28 ± 0.52 | 17.50 ± 0.50 | 4.05 |
| **SUPER H2^®^** | 16.84 ± 0.73 | 17.68 ± 0.63 | 17.50 ± 0.50 | 4.05 |

Data are expressed as the mean ± standard deviation (n=5). H_2_, molecular hydrogen.

**Supplementary Figure S1.** Changes in the exhaled hydrogen concentration over time in each of the 10 participants


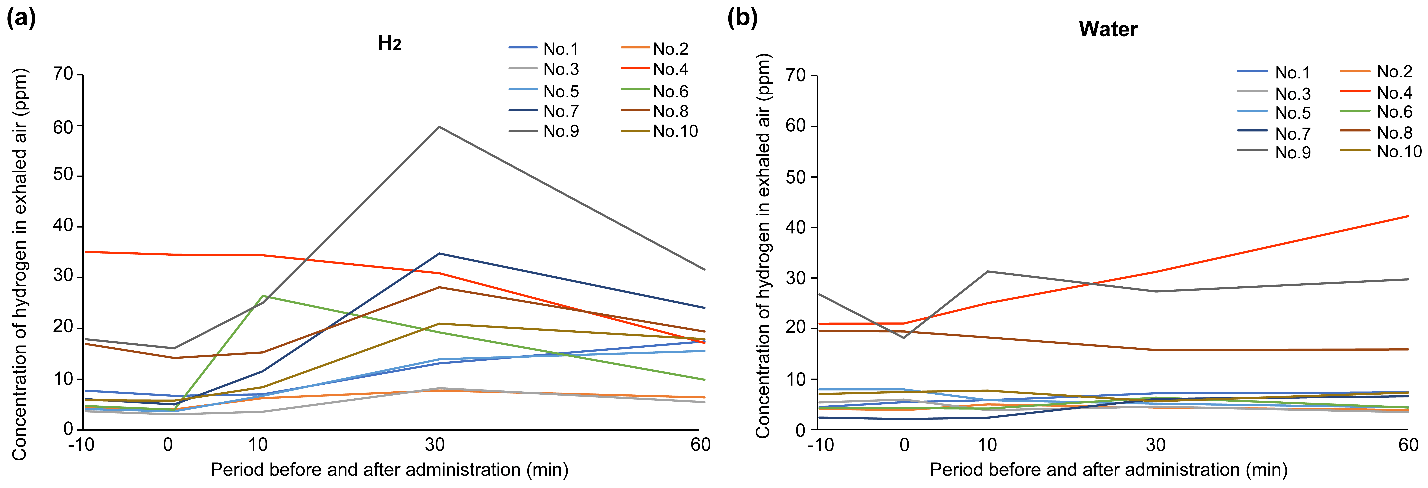


H_2_: molecular hydrogen, No: participant number.
